# Lumican – Derived Peptides Inhibit Melanoma Cell Growth and Migration

**DOI:** 10.1371/journal.pone.0076232

**Published:** 2013-10-02

**Authors:** Katarzyna Pietraszek, Stéphane Brézillon, Corinne Perreau, Maria Malicka-Błaszkiewicz, François-Xavier Maquart, Yanusz Wegrowski

**Affiliations:** 1 Laboratoire de Biochimie Médicale et de Biologie Moléculaire, CNRS FRE 3481, Université de Reims-Champagne-Ardenne, Reims, France; 2 Department of Cell Pathology, Faculty of Biotechnology, University of Wrocław, Wrocław, Poland; 3 CHU de Reims, Reims, France; University of Patras, Greece

## Abstract

Lumican, a small leucine-rich proteoglycan of the extracellular matrix, presents potent anti-tumor properties. Previous works from our group showed that lumican inhibited melanoma cell migration and tumor growth *in vitro* and *in vivo*. Melanoma cells adhered to lumican, resulting in a remodeling of their actin cytoskeleton and preventing their migration. In addition, we identified a sequence of 17 amino acids within the lumican core protein, named lumcorin, which was able to inhibit cell chemotaxis and reproduce anti-migratory effect of lumican *in vitro*. The aim of the present study was to characterize the anti-tumor mechanism of action of lumcorin. Lumcorin significantly decreased the growth in monolayer and in soft agar of two melanoma cell lines – mice B16F1 and human SK-MEL-28 cells – in comparison to controls. Addition of lumcorin to serum free medium significantly inhibited spontaneous motility of these two melanoma cell lines. To characterize the mechanisms involved in the inhibition of cell migration by lumcorin, the status of the phosphorylation/dephosphorylation of proteins was examined. Inhibition of focal adhesion kinase phosphorylation was observed in presence of lumcorin. Since cancer cells have been shown to migrate and to invade by mechanisms that involve matrix metalloproteinases (MMPs), the expression and activity of MMPs were analyzed. Lumcorin induced an accumulation of an intermediate form of MMP-14 (~59kDa), and inhibited MMP-14 activity. Additionally, we identified a short, 10 amino acids peptide within lumcorin sequence, which was able to reproduce its anti-tumor effect on melanoma cells. This peptide may have potential pharmacological applications.

## Introduction

Melanoma metastasis is a complex series of coordinated events, which starts from colonization of nearest ganglions and distant tissues to form secondary sites of tumor growth [1]. One of the first steps in this process is the degradation of basement membrane components and extracellular matrix (ECM) by matrix metalloproteinases (MMPs) [2,3]. Thus, increased expression of MMPs has been linked to tumor progression and invasion [4]. Histopathological analysis of human tumor samples demonstrated that membrane type metalloproteinase -1 (MMP-14) is expressed in melanoma cells and localized to tumor cells at the invasive front of primary tumors [4]. MMP-14 is particularly localized in the invadopodia of the leading edge of migrating cells [5]. Overexpression of MMP-14 was shown to promote selective invasion and growth of malignant melanoma *in vivo* [6]. MMP-14 itself can degrade extracellular matrix macromolecules, either directly or by activating other MMPs, like pro-MMP-2 [7].

Following degradation of epidermal-dermal junction, melanoma cells migrate across extracellular matrix of the dermis. ECM is composed of molecules interacting with one another, including fibrous proteins, proteoglycans and hyaluronan, whereas MMPs are involved in its remodeling [8].

Small Leucine Rich Proteoglycans (SLRPs) are abundant components of dermis ECM. The SLRP family is made up of several structurally and functionally related members including lumican, decorin, biglycan, fibromodulin, which are thought to guide matrix assembly and organization through protein: protein and/or protein: carbohydrate interactions [9]. Lumican, like other members of this group, possesses 11 leucine rich repeats (LRR) [10], which contain a 11 amino acid motif [11]. LRR motif participates in collagen assembly process [12].

Apart from its structural function in the control of collagen fibril assembly, SLRPs, particularly lumican and decorin, can regulate tumor cell behavior [13]. Lumican was shown to inhibit melanoma progression *in vivo* with a concomitant decrease of cyclin D1 expression and to induce and/or increase apoptosis [14]. Moreover, lumican was able to decrease melanoma cell lung metastasis [15]. α2β1 integrin was characterized as a lumican receptor on tumor cells [16]. In the presence of lumican, reorganization of actin cytoskeleton and destabilization of focal adhesion complexes with cytosolic accumulation of vinculin were observed [17,18]. Lumican was also able to inhibit angiogenesis, down-regulating the proteolytic activity associated with surface membranes of endothelial cells [19]. It was shown to alter MMP-14 expression and activity in mesenchymal stem cells [20]. Conversely, lumican can be degraded by MMP-14, revoking its anti-tumor properties which depend on intact native molecule [21].

Previous works from our laboratory identified a sequence of 17 amino acids (aa) within the leucine-rich repeat 9 [22], which was able to reproduce anti-migratory effect of lumican *in vitro* by inhibiting cell chemotaxis. This sequence of the lumican core protein was named lumcorin. This study reports the characterization of anti-migratory mechanisms of lumcorin and the design of a short, 10 amino acid peptide (L9M) which is able to reproduce this anti-tumor effect on melanoma cells.

## Materials and Methods

### Reagents

Lumcorin, the peptide corresponding to the LRR9 motif of lumican (SSLVELDLSYNKLKNIP), L9M, the 10 aa peptide from lumcorin central part (underlined), the scrambled (SCR) peptides (lumcorin SCR – LPSVSILEKLYNNLSKD, L9M SCR – SLELDLNKYK) and the corresponding peptides from decorin (LRR9 DCN – PHLRELHLDNNKLTRVP) and fibromodulin (Fmod LRR9 – SSLLELDLSYNQLQKIP) were obtained from Genscript (Piscataway, USA). In all experiments, 100µM concentration of these peptides was used. The following primary antibodies were used: mouse monoclonal anti-human pFAK (pY397) (BD Biosciences, Bedford, MA, USA), rabbit polyclonal anti-mouse total FAK, rabbit polyclonal antibody directed against the hinge region of human MMP-14 (Abcam, Cambridge, UK), and goat anti-human actin (Santa Cruz Biotechnology, Heidelberg, Germany). The corresponding secondary antibodies conjugated to horseradish peroxidase were purchased from GE Healthcare (Orsay, France).

### Cell culture and cell growth assay

Murine B16F1 melanoma cells (CRL-6323™) and SK-MEL-28 (HTB-72™) human malignant melanoma cells, were obtained from ATCC. Cells were cultured in DMEM medium in standard conditions [14]. In all experiments, cell viability was greater than 95%, as assessed by trypan blue exclusion test.

Cell growth was determined using MTT test on 96-well plates for 1×10^4^ cells/well [17]. Cells were grown for 24, 48 and 72 h in the presence of 100µM lumcorin or L9M or their corresponding scrambled peptides. Cell growth was then analyzed using 3-[4,5-dimethylthiazol-2-yl]-2,5-diphenyltetrazolium bromide (MTT, Sigma). For this purpose, cells were incubated with culture medium supplemented with 0.5 mg/ml MTT for 3h at 37°C. MTT solution was then replaced by DMSO and absorbance at 560 nm was measured.

### Anchorage-independent growth in soft agar

Soft agar growth assays [14] were carried out in 12-well plates. Each well contained the following layers: a bottom layer 0.9% agar, a middle layer 0.3% agar containing the cell suspension (1.2×10^3^cells/well) and a top layer 0.9% agar. The layers were covered by complete culture medium. When needed, 100µM lumcorin or L9M or their scrambled peptides were added to the middle layer of agar. After 7 days of culture, growth medium was replaced with complete medium with 100µM peptides. After additional 7 days of cell culture, the number of colonies was counted in triplicate and diameter of 100 colonies was measured using Image Tool software (UTHSCSA Image Tool for Windows version 3.0, San Antonio, TX).

### Migration assay

Migration assay was performed using culture-inserts (Biovalley, Marne-la-Vallée, France). Cells were seeded on 24-well plates in culture-inserts with 3x10^4^ cells per chamber in 70 µL of complete cell culture medium. After 24h of incubation at 37°C, the culture inserts were removed, cells were rinsed with PBS and the wells were filled with 1 mL of serum-free cell culture medium supplemented with 100µM lumcorin or L9M and their scrambled peptides. Cell motility was followed using an inverted microscope (Axiovert 200M; Zeiss, Oberkoken, Germany) equipped with a transparent environmental chamber (Climabox; Zeiss) with 5% (v/v) CO_2_ in air at 37°C. The microscope was driven by the Metamorph^®^ software (Roper Scientific, Évry, France). The cell position was recorded with a charge-coupled device camera (CoolsnapHQ: Roger Scientific) for 48h at 30 min intervals. Cell migration from 4 fields per insert, 3 replicate inserts for each condition, was analyzed using Image Tool software by quantification of cell-free surface and next calculated as percent of filled area. See File S1 for more details.

### MMP-14 activity assay

The MMP-14 activity was measured using a SensoLyte^®^ 520 MMP-14 Assay Kit (AnaSpec, San Jose, USA) in cell lysates (50µg) after 48h of incubation with peptides. To determine direct effect of lumcorin on MMP-14 activity, B16F1 cell protein extract (50µg) or the recombinant catalytic domain (Ala 21-Gly 284) of human MMP-14 (Anaspec, Seraing, Belgium) (5ng) were pre-incubated 15 min or 60 min with 100µM lumcorin or L9M peptide or their corresponding scrambled peptides or DCN LRR9 or Fmod LRR9. The activity of MMP-14 in samples was measured according to the protocol of the supplier.

### Western blotting

Total cell proteins (30µg) were subjected to electrophoresis in a 0.1% SDS polyacrylamide gel. Proteins were transferred onto Hybond-P PVDF membranes (GE Healthcare, Orsay, France) by electroblotting and detected using specific antibodies. The bands were revealed by the ECL Prime Chemiluminescence Detection reagent (GE Healthcare) as indicated by the manufacturer. The chemiluminescence signal was captured using a ChemiDoc™ MP Imaging System (Bio-Rad) adjusting the exposure time so that all dots were below pixel saturation.

### Statistical analysis

Results were expressed as mean ± S.D. Statistical significance between groups was assessed by one way analysis of variance (ANOVA) with Bonferroni correction using SPSS Statistics software. The *p* value < 0.05 was considered statistically significant.

## Results

### Lumcorin inhibits melanoma growth and motility

**Figure 1:**
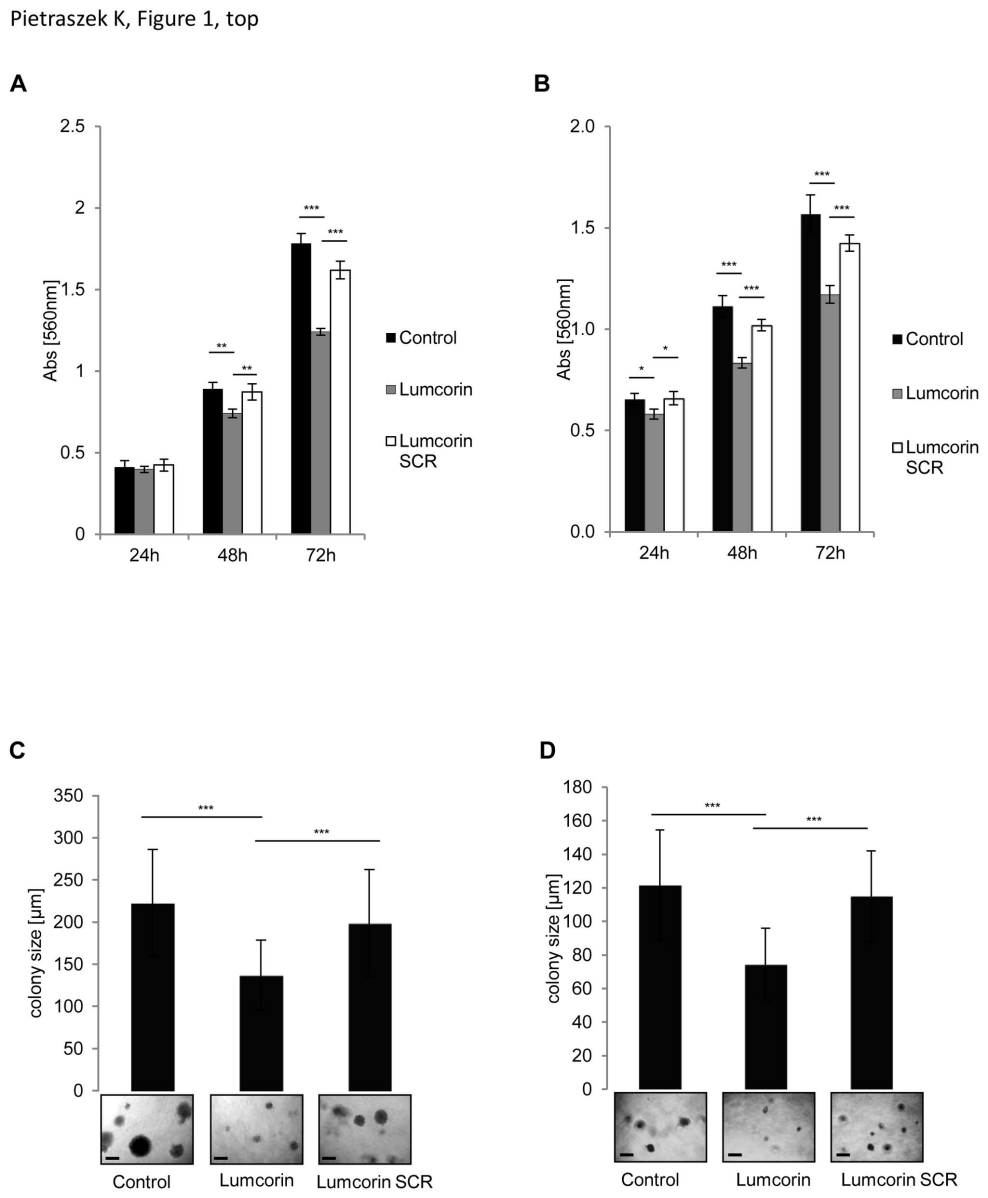
Lumcorin decreases melanoma growth. Cell growth assay (A, B) and colony formation assay (C, D) of B16F1 cells (A, C) and SK-MEL-28 cells (B, D). For growth studies, cells were grown for 24, 48 and 72h in presence of 100 µM lumcorin or its scrambled (SCR) peptide. Cell growth was measured by MTT colorimetric test at 560nm, as described in Materials and Methods. Results were reported as mean ± S.D of sextuplicate values from three independent experiments. For colony formation, 1.2x10^3^cells were cultured in 0.3% agar for 14 days in presence of 100µM lumcorin or scrambled peptide as described in Materials and Methods. Representative images of cell colonies are displayed at the inserts. The quantification of the colony diameter was done using Image Tool software. Graphs represent the mean size of 100 colonies ± S.D from three independent experiments (*, *p*< 0.05, **, *p*<0.01***, *p*<0.001). Scale bar in the inserts: 200µm.

Previous studies described anti-proliferative and anti-migratory properties of lumican [14]. A sequence responsible for inhibition of melanoma cell chemotaxis was determined and named lumcorin [22]. To confirm whether lumcorin was able to reproduce lumican effects, the proliferation and spontaneous motility of melanoma cells were tested. Two melanoma cell lines were selected: B16F1, a tumorigenic murine melanoma cell line and SK-MEL-28, a human skin malignant melanoma cell line. After 48 and 72h of incubation of the cells with lumcorin, a significant inhibition of cell growth was observed in comparison to scrambled lumcorin peptide in B16F1 cells (Figure 1A). Similar results were observed at 24, 48 and 72h in SK-MEL-28 cells (Figure 1B). After 72h, the inhibition of cell growth was of 25% for B16F1 cells and 20% for SK-MEL-28 cells. To measure the effect of lumcorin on cell proliferation in a semi-solid culture medium, the anchorage-independent growth of B16F1 and SK-MEL-28 cells in soft agar was investigated. The sizes of B16F1 and SK-MEL-28 colonies were decreased by 40% (Figure 1C and D) after 14 days of cell culture in the presence of lumcorin in comparison to scrambled lumcorin peptide.

**Figure 2:**
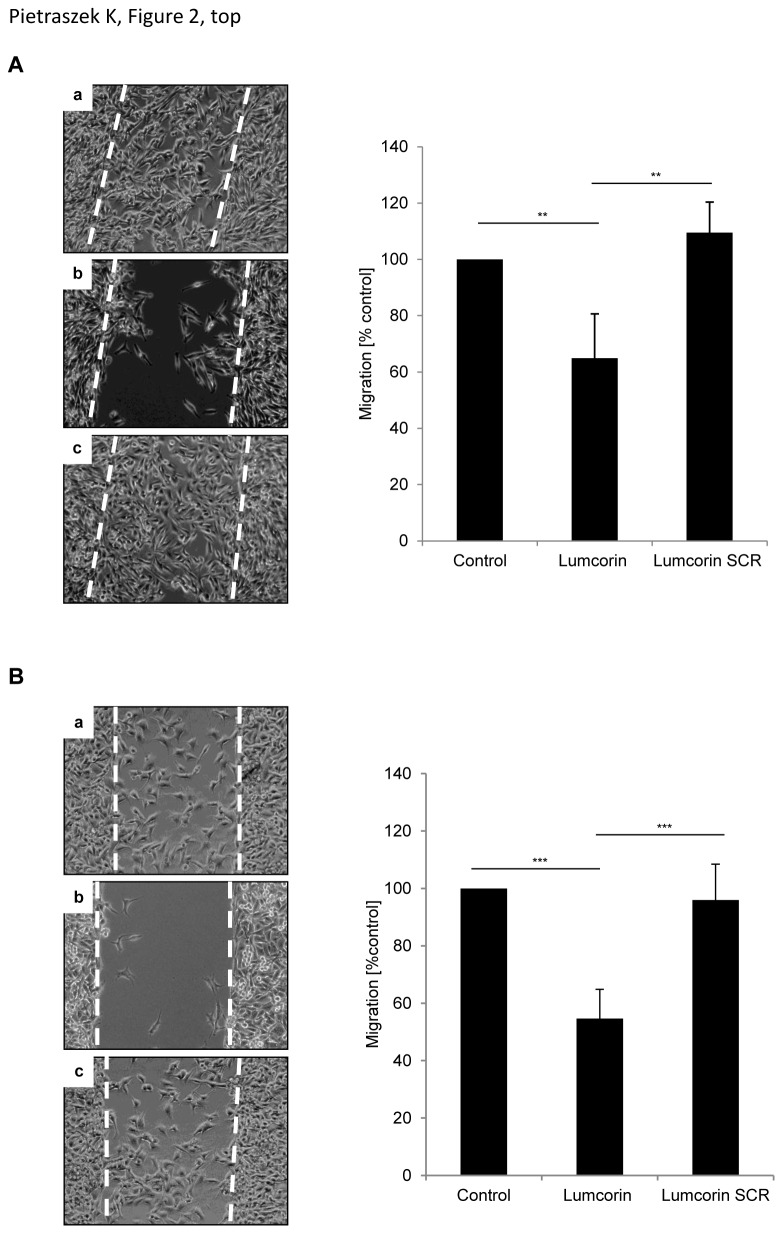
Lumcorin inhibits melanoma motility. Migration of B16F1 cells (A) and SK-MEL-28 cells (B) in the presence of lumcorin. Cells were plated on 24-well plate, 3x10^4^ cells per chamber of culture-insert. After 24h incubation, the culture-inserts were withdrawn and migration was monitored for 48h by computer-assisted phase contrast videomicroscopy as described in Materials and Methods. Representative images of cell positions after 48h of migration in the absence of peptide (a), in presence of 100µM lumcorin (b) or of its scrambled peptide (c) are displayed on left panels. Migration was quantified as percent of area colonized by cells. Graphs represent the mean value ± S.D calculated from 4 microscopic fields per insert. The experiment was done in triplicate (**, *p*<0.01, ***, *p*<0.001).

To investigate whether lumcorin altered the spontaneous motility of melanoma cells, cell migration assay was performed. In control conditions, as well as in presence of the scrambled lumcorin peptide, B16F1 cells completely closed the wound after 48h. Lumcorin (100µM) decreased the spontaneous B16F1 cell migration by nearly 40% (Figure 2A) and SK-MEL-28 cell migration by 45% (Figure 2B) as compared to control cells. Moreover, 10µM lumcorin was able to significantly inhibit B16F1 cell migration (Figure S1A), but to a lesser extent. In both cell lines, the corresponding scrambled lumcorin peptide had no effect on cell motility. These results show that lumcorin, similarly to lumican, is able to decrease growth of different melanoma cell lines *in vitro* and to inhibit their spontaneous migration.

### Lumcorin alters the expression and activity of MMP-14

**Figure 3:**
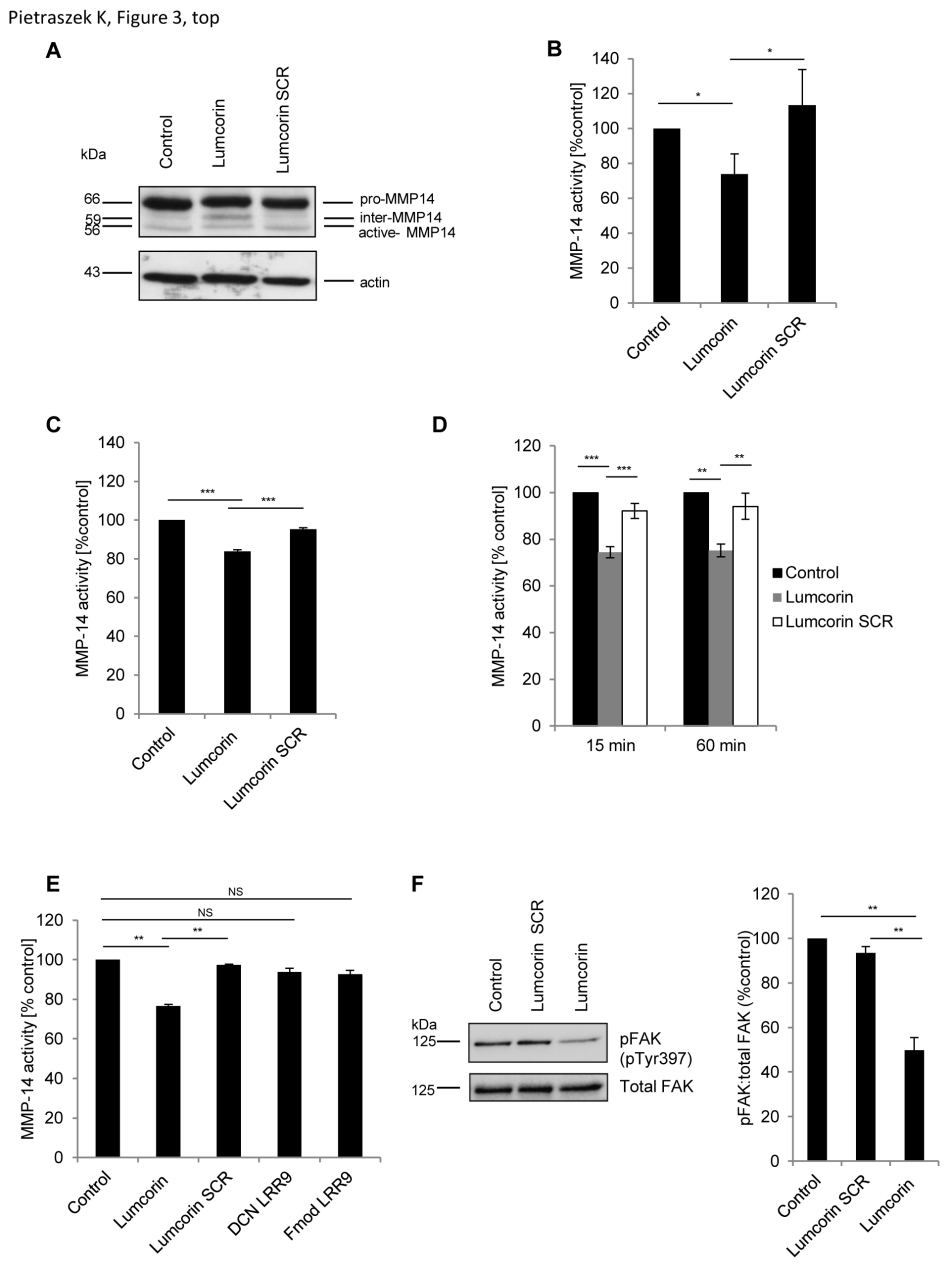
Lumcorin alters the expression and activity of MMP-14 and inhibits FAK phosphorylation at tyrosine 397. (A) Expression of MMP-14 in B16F1 cells incubated 48h with 100µM lumcorin or scrambled peptide was analyzed by Western immunoblotting using polyclonal rabbit antibody and probing with anti-β-actin. (B, C) Activity of MMP-14 in B16F1 (B) and SK-MEL-28 (C) cells incubated 48h in the presence of 100µM lumcorin or its scrambled peptide, measured using fluorimetric SensoLyte® 520 MMP-14 Assay Kit as described in Materials and Methods. (D) Activity of MMP-14 in B16F1 cell extract pre-incubated 15 min or 60 min before assay with 100µM lumcorin or scrambled peptide. (E) Recombinant human MMP-14 activity pre-incubated 15 min with 100µM lumcorin or its scrambled peptide or DCN LRR9 or Fmod LRR9. Data are presented as mean ± S.D from three independent experiments. (F) Phospho-FAK (pY397) and total FAK expression in B16F1 cells incubated 15 min with 100µM lumcorin or scrambled peptide analyzed by Western immunoblotting using monoclonal mouse antibody against pFAK (pY397) and probing with polyclonal rabbit anti-total FAK antibody. After densitometric analysis of the intensity of the bands, the resulting ratio of pFAK to total FAK intensity was calculated and presented on the graph. Data are presented as mean values ± S.D from three independent experiments (*, *p*<0.05; **, *p*<0.01; ***, *p*<0.001; NS, no significant difference).

Since cancer cells have been shown to migrate and to invade ECM by mechanisms that involve matrix metalloproteinases, the expression of MMP-14 in B16F1 cells was analyzed using real-time PCR and Western immunoblotting. The level of MMP-14 transcript was found to be similar between cells incubated with lumcorin and control cells (not shown). Inactive pro-MMP-14 (~66kDa) and active form of MMP-14 (~56kDa) were detected in B16F1 cells, in control cells and cells incubated for 48h with scrambled lumcorin peptide (Figure 3A) by Western immunoblotting. However, in presence of lumcorin, cells expressed an additional intermediate form of MMP-14 (~59kDa), which was barely detectable in control conditions.

MMP-14 is produced as an inactive zymogen that is activated by furin-like convertases, which cleave at the motif located between the pro-peptide and the catalytic domain [23]. No alteration of the furin activity was observed in B16F1 cells treated for 48h with lumcorin in comparison to scramble peptide or control without peptide (Figure S2). Immunolocalization using the MMP-14 antibody showed that after 48h in presence of the lumcorin SCR, MMP-14 was localized at the cell surface and in the cytoplasm of B16F1 cells, and in about 50% of cells, at the migration front and in some focal adhesions (Figure S3). In contrast, in cells treated with lumcorin, MMP-14 distribution was strongly decreased at the migration front and in focal adhesions with only about 20% of cells exhibiting positive spots (likely focal adhesions).

Subsequently, MMP-14 activity was tested in B16F1 and SK-MEL-28 cells cultured in presence of lumcorin for 48h. MMP-14 activity decreased when both cell lines were incubated with lumcorin (Figure 3B and C). To determine whether lumcorin directly regulated MMP-14 activity, B16F1 cell protein extracts were pre-incubated for 15 and 60 min with lumcorin or its scrambled peptide before MMP-14 activity assay. After 15 min of pre-incubation with lumcorin, MMP-14 activity was decreased by 20% in comparison to non-treated lysates or lysates pre-incubated with scrambled peptide (Figure 3D). Longer time (60min) of pre-incubation showed the same effect of lumcorin. This result suggests that lumcorin may directly interact with the enzyme. To clarify this point, MMP-14 activity was measured using recombinant human MMP-14 pre-incubated 15 min with lumcorin or the corresponding scrambled peptide. Lumcorin significantly inhibited MMP-14 activity in comparison to control or with scrambled peptide conditions (Figure 3E). To determine whether the observed effect is specific for lumcorin, corresponding peptides from decorin (DCN LRR9) and fibromodulin (Fmod LRR9) were tested. In presence of the corresponding LRR9 motifs from two other SLRPs no significant decrease of recombinant MMP-14 activity was observed. This result indicates that lumcorin may affect directly but also indirectly MMP-14 activity without affecting its mRNA steady state level.

Cell migration through ECM is also regulated by the expression and activity of other matrix metalloproteinases. However, lumcorin did not influence MMP-2 nor MMP-9 activities in B16F1 and SK-MEL-28 cells (not shown).

### Lumcorin inhibits FAK phosphorylation at tyrosine 397

The inhibition of melanoma cell migration by lumican was correlated with the inhibition of the phosphorylation of focal adhesion kinase (FAK) [16]. To compare if lumcorin could act through similar mechanism, FAK phosphorylation status was studied by Western immunoblotting in B16F1 cells (Figure 3F). The level of phosphorylation of FAK at tyrosine 397 was similar when the cells were preincubated with scrambled peptide in comparison to not treated cells. On the contrary, after 15 min incubation of B16F1 cells with lumcorin, the pFAK (pY397)/FAK ratio was decreased by 40% in comparison to both controls. In parallel, the analysis of the cytoskeleton showed no significant alteration of the actin network (Figure S4). This result suggests that lumcorin may also inhibit melanoma cell migration by involvement of FAK phosphorylation.

### Characterization of the minimal active sequence from lumcorin

To determine the minimal active sequence of lumcorin, a 10 amino acid peptide (L9M) reproducing the central amino acid sequence of lumcorin (ELDLSYNKLK) was designed. To examine whether L9M peptide is able to reproduce effects of lumcorin, its influence on melanoma cell proliferation, anchorage-independent growth in soft agar and spontaneous cell migration were tested.

**Figure 4:**
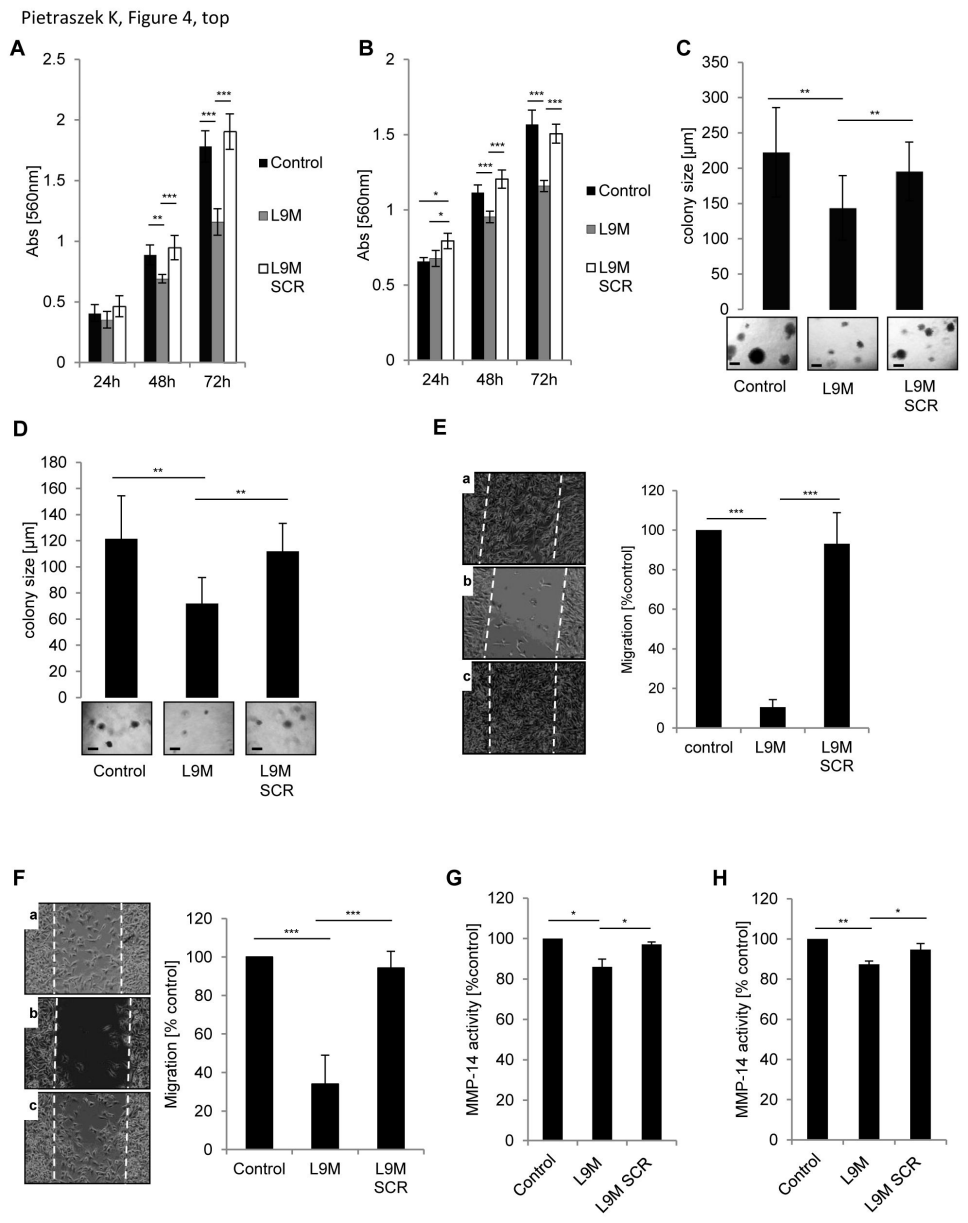
Characterization of lumcorin derived L9M peptide. Cell growth assay (A, B) and colony formation assay (C, D) of B16F1 cells (A, C) and SK-MEL-28 cells (B, D) cultured in the presence of 100 µM L9M or appropriate scrambled peptide (L9M SCR). Cell growth was measured as described in Figure 1. Results were reported as mean ± S.D of sextuplicate values from three independent experiments. For colony formation 1.2x10^3^cells were cultured in 0.3% agar for 14 days in presence of 100µM L9M or L9M SCR as described in Materials and Methods. Representative images of cell colonies are displayed at the inserts. The quantification of the colony diameter was done using Image Tool software. Graphs represent the mean size of 100 colonies ± S.D from three independent experiments. Scale bar in the inserts: 200µm. (E, F) Migration of B16F1 cells (E) and SK-MEL-28 cells (F) in presence of L9M peptide. Cells were plated on 24-well plate at with 3x10^4^ cells per chamber of culture-insert. After 24h of incubation, the culture-inserts were withdrawn and migration was monitored for 48h by computer-assisted phase contrast videomicroscopy. Representative images of cell positions after 48h of migration in the absence of peptide (a), in presence of 100µM L9M peptide (b) or its scrambled peptide (c) are displayed on left panels. Migration was quantified as percent of area colonized by cells. Graphs represent the mean value ± S.D calculated from 4 microscopic fields per insert. (G, H) Effect of L9M peptide on MMP-14 activity in B16F1 cells (G) and SK-MEL-28 cells (H). MMP-14 activity in cells incubated 48h in the presence of 100µM L9M or its scrambled peptide was measured using fluorimetric SensoLyte® 520 MMP-14 Assay Kit. The experiment was done in triplicate (*, *p*<0.05; **, *p*<0.01; ***, *p*<0.001).

After 48 and 72h of incubation in the presence of L9M peptide, the growth in monolayer of B16F1 (Figure 4A) and SK-MEL-28 cells (Figure 4B) was inhibited when compared to scrambled L9M peptide. Similarly, the anchorage-independent growth of B16F1 and SK-MEL-28 cells in soft agar was analyzed. Interestingly, after 14 days of cell culture in presence of L9M peptide, the number of colonies formed by B16F1 cells was similar in each condition, but we observed a significantly decreased size of colonies in the presence of L9M peptide in comparison to scrambled L9M peptide (Figure 4C and D).

L9M peptide also affected the spontaneous motility of B16F1 and SK-MEL-28 melanoma cells. The cell migration assay showed that 100 µM L9M peptide drastically decreased the migration of B16F1 cells by 90% (Figure 4E) and of SK-MEL-28 cells by 60% (Figure 4F) as compared to the control. Moreover, 1 and 10µM L9M were able to significantly inhibit B16F1 cell migration (Figure S1B), but to a lesser extent. Scrambled L9M peptide had no effect on the spontaneous migration of both cell types.

To check whether the mechanisms of inhibition of migration of melanoma cells by L9M peptide were similar to that of lumcorin, the activity of MMP-2, MMP-9 and MMP-14 was examined in B16F1 and SK-MEL-28 cell line incubated for 48h with L9M peptide or its corresponding scrambled peptide. No significant change of the activity of MMP-2 and MMP-9 by cells treated with L9M peptide was observed (not shown). However, in both type of cells, L9M peptide induced slight but significant decrease of MMP-14 activity (Figure 4G, H).

These preliminary data suggest that the L9M 10 aa peptide of lumican LRR9 can inhibit growth and migratory properties of melanoma cells.

## Discussion

Tumor cell migration is an important process during melanoma progression, leading to invasion and metastases. ECM macromolecules are involved in the control of this process by inhibiting cell propagation to adjacent lymphatic nodes during the first steps of metastatic process [24]. The ECM component, lumican, may be considered as an anti-tumor molecule, since it was shown to decrease melanoma progression *in vivo* [14]. *In vitro*, it was demonstrated that the inhibition of melanoma cell chemotactic migration by lumican is due to a specific sequence of the core protein located in the LRR9 domain. This sequence was named lumcorin [22]. In this study, we characterized some properties of lumican – derived peptides on anchorage dependent and independent growth and spontaneous migratory properties of melanoma cells.

Lumican is able to significantly inhibit the anchorage-independent growth of melanoma cells in soft agar [14]. Colony formation by HeLa cells was also suppressed by the expression of lumican [21]. This suppression was rescued by coexpression of MMP-14 and lumican, leading to lumican degradation. However, lumcorin was able to reproduce the anti-proliferative effects of lumican. After 14 days of culture in soft agar in the presence of lumcorin, colonies of the two melanoma cell lines presented a 40% decreased size. Moreover, lumcorin was able to inhibit B16F1 cells and SK-MEL-28 cell growth in monolayer culture, which was not observed in the case of lumican [14,17]. Lumican was reported to inhibit melanoma and endothelial cell migration [18,19,25]. In this paper, we showed that lumcorin is not only able to decrease the chemotactic migration of melanoma cells [22], but also to inhibit spontaneous motility of melanoma cells. Lumcorin, similarly to lumican, acts on two fundamental processes connected to melanoma progression: cell growth and migration.

Tumor progression is associated with ECM degradation and proteoglycans play a major role in the control of this process. Cancer cells have been shown to migrate and invade by mechanisms that involve matrix metalloproteinases. It was shown that lumican influences the expression and activity of MMP-9 and MMP-14 [19,20]. However, the expression of MMP transcripts was similar between cells incubated with lumcorin and control cells. On the other hand, after 48h incubation of the cells with lumcorin, MMP-14 activity significantly decreased in B16F1 and SK-MEL-28 cells. Moreover, the inhibition of MMP-14 activity that we observed after incubation of recombinant enzyme with lumcorin suggests that lumcorin may directly interact with MMP-14 without influencing its expression. Since the recombinant catalytic domain of human MMP-14 was used in the assay, this data suggest that lumcorin binds to the catalytic domain of MMP-14, inhibiting its activity. Moreover, in the presence of lumcorin, cells expressed, in addition to pro-MMP-14 and mature MMP-14, an intermediate ~59kDa form of MMP-14. A similar intermediate form of MMP-14 was also detected in mesenchymal stem cells treated with lumican [20]. It was also described in thymocytes and was suggested to be inactive [26]. MMP-14 is produced as an inactive zymogen that is activated by furin-like convertases, which cleave at the motif located between the pro-peptide and the catalytic domain [23]. However, lumcorin did not alter the activity of furin-like convertases in B16F1 cells. Therefore, this induction of expression of an inactive intermediate form of MMP-14 suggests an abnormal maturation of MMP-14 processing.

A relocation of MMP-14 from the migration front and focal adhesion was observed in presence of lumcorin. This result suggests an abnormal MMP-14 trafficking. This could also contribute to the inhibition of cell migration by lumcorin.

The hemopexin domain of MMP-14 participates in dimerization of the enzyme on the cell surface [27]. This dimerization of MMP-14 is required for pro-MMP-2 activation and then regulates cellular invasiveness. Lumcorin does not alter the activation of MMP-2 in B16F1 and SK-MEL-28 cells, suggesting that this peptide may not affect dimerization of MMP-14.

These results indicate that lumcorin may affect directly MMP-14 activity or its maturation and/or trafficking, which could partially explain the inhibition of the migration properties of melanoma cells in the presence of lumcorin. MMP-14 plays an important role in cell migration, not only by regulating the activity or expression of downstream MMPs but also by processing and activating migration-associated molecules such as integrins or CD44, and a variety of intracellular signaling pathways such as MAPK, FAK, Src and Rac [28–31].

Lumican effect was characterized by an increased cell adhesion mediated by β1 integrin expression [25]. It was shown that melanoma cells, when in contact with a lumican core protein substratum, exhibited a change in the distribution of the β1 integrin subunit on cell membrane. Concomitantly, a reorganization of actin stress fibers and a significant decrease in vinculin immunostaining at focal adhesion complexes were observed [17,18]. A significant decrease of the ratio pFAK/FAK was also shown in presence of recombinant human lumican [16,18]. In presence of lumcorin, an inhibition of FAK tyrosine 397 phosphorylation was also observed. The ratio pFAK/FAK was decreased by 50% in comparison to control. On the contrary, no rearrangement of actin filament organization or reorganization of focal adhesion complexes was detected. Therefore, the decreased FAK phosphorylation and the inhibition of MMP-14 activity induced by lumcorin in melanoma cells might explain, at least in part, the anti-migratory effect of this peptide.

Alignment of lumcorin with the corresponding sequence of four human SLRPs (lumican, fibromodulin, decorin and biglycan) showed 54% of conserved amino acids within this motif. However, only lumcorin was able to significantly inhibit recombinant MMP-14 activity in comparison to corresponding LRR9 of decorin and fibromodulin, suggesting that observed effect is specific for this peptide. The 10 aa central part of lumcorin (L9M) was characterized by the highest identity. For that reason, we hypothesized that L9M was the minimal active sequence. Interestingly, the corresponding motif of fibromodulin differs from the lumican – derived L9M peptide sequence by only two lysine at positions 265 and 267, which are replaced by glutamine in fibromodulin.

Growth of melanoma cells in the presence of L9M peptide was similarly affected in comparison to lumcorin. This L9M peptide inhibited B16F1 cell and SK-MEL-28 cell motility in a very efficient way. Migration assay showed that L9M peptide drastically decreased the spontaneous migration of B16F1 cells and of SK-MEL-28 cells, whereas lumcorin inhibited cell migration in a less efficient way. This effect can be partially explained by the slight, but significant inhibition of MMP-14 activity induced by L9M peptide. However, these results indicate that there might be other mechanisms of action, which still remain unclear. It was shown that the central fragment of decorin LRR5 was also more active in inhibition of angiogenesis than the full-length LRR [32]. Similarly, the central 12 amino acid region of decorin LRR5 was demonstrated to inhibit endothelial cell tube formation up to 1000 times more potently than the full length LRR5. Recently, several peptides derived from ECM components were shown to modulate the growth or the invading properties of tumor cells. For instance, the NC1 domains of the α3(IV) chain and α4(IV) chain of type IV collagen inhibit the invasive potential of several tumor cell lines by decreasing their proliferation and migration [33–35]. Other peptides derived from connective tissue glycoproteins, like anastelin, may also decrease tumor growth [36].

Altogether, we propose two simultaneous mechanisms by which lumcorin is able to inhibit melanoma cell migration: inhibition of phosphorylation of specific proteins and decrease of MMP-14 activity. Further studies are necessary to clarify the mechanisms of action of L9M peptide, which seems to be the minimal active sequence from lumican. The presented data suggest that lumcorin and the lumican-derived 10 amino acid L9M peptide, which possess strong anti-proliferative and anti-migratory effects, might have therapeutic applications.

## Supporting Information

Figure S1 **Dose - dependent effect of lumcorin and L9M peptide on the migration of B16F1 cells.** B16F1 cells were plated on 24-well plate, 3x10^4^ cells per chamber of culture-insert. After 24h incubation, the culture-inserts were withdrawn and migration was monitored in a presence of 1, 10, 100µM lumcorin (A) or L9M peptide (B) for 48h by computer-assisted phase contrast videomicroscopy as described in Materials and Methods. Migration was quantified as percent of area colonized by cells. Graphs represent the mean value ± S.D calculated from 4 microscopic fields per insert. The experiment was done in triplicate (*, *p*<0.05, **, *p*<0.01, ***, *p*<0.001).
(TIF)

Figure S2 **Lumcorin has no effect on furin convertase activity.** Furin-like enzyme activity in B16F1 cells incubated 48h in the presence of 100µM lumcorin or its scrambled peptide, measured using fluorogenic substrate (Pyr-Arg-Thr-Lys-Arg-AMC trifluoroacetate salt) as described in Materials and Methods.
(TIF)

Figure S3 **Lumcorin alters MMP-14 distribution.** MMP-14 immunolocalization in B16F1 cells after 48h incubation in presence of 100µM lumcorin SCR (a), lumcorin (b). MMP-14 was visualized by confocal microscopy using an antibody directed against the hinge region of MMP-14. Arrowheads indicate the migration front and empty arrowheads focal adhesions. Scale bar: 20 µm.
(TIF)

Figure S4 **Lumcorin and L9M peptide do not induce a rearrangement of actin filament organization.** Actin cytoskeleton distribution in B16F1 (a-d) and SK-MEL-28 (e-h) cells after 48h incubation in presence of 100µM lumcorin SCR (a,e), lumcorin (b,f), L9M SCR (c,g) and L9M (d,h). Filamentous actin was visualized under a fluorescence microscope after staining with Alexa Fluor ^®^488-conjugated phalloidin. Scale bar: 20 µm.
(TIF)

File S1 **Supporting Materials and Methods.**
(DOC)
