# Supplementary figures and images for: Lumican – Derived Peptides Inhibit Melanoma Cell Growth and Migration
Source: PLoS One. 2013 Oct 2;8(10):e76232. doi: 10.1371/journal.pone.0076232 (PMC3788744; doi:10.1371/journal.pone.0076232)

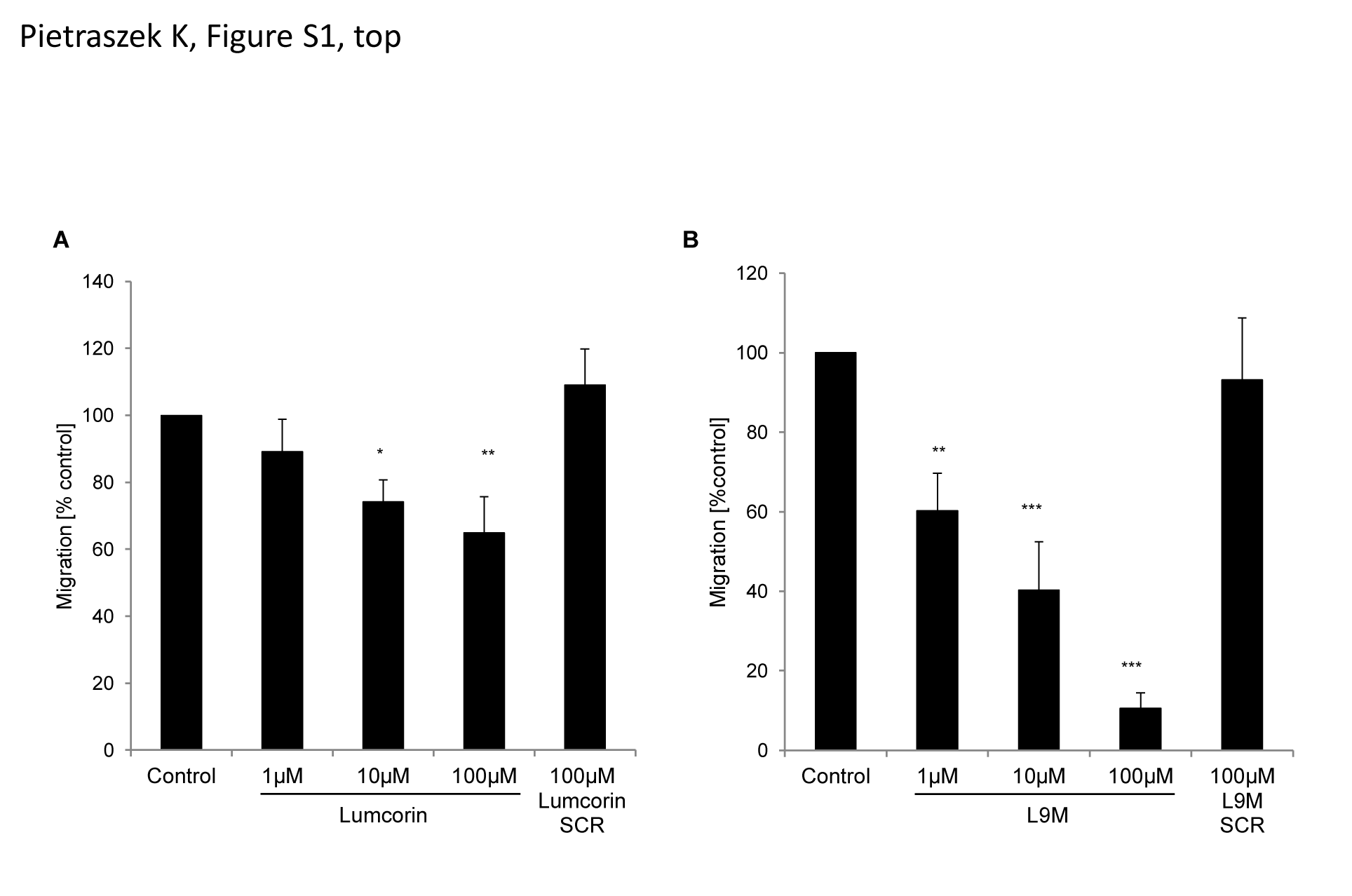

Supplement: Figure S1 — Dose - dependent effect of lumcorin and L9M peptide on the migration of B16F1 cells. B16F1 cells were plated on 24-well plate, 3x10^4^ cells per chamber of culture-insert. After 24h incubation, the culture-inserts were withdrawn and migration was monitored in a presence of 1, 10, 100µM lumcorin (A) or L9M peptide (B) for 48h by computer-assisted phase contrast videomicroscopy as described in Materials and Methods. Migration was quantified as percent of area colonized by cells. Graphs represent the mean value ± S.D calculated from 4 microscopic fields per insert. The experiment was done in triplicate (*, p<0.05, **, p<0.01, ***, p<0.001). (TIF) [file pone.0076232.s001.tif]

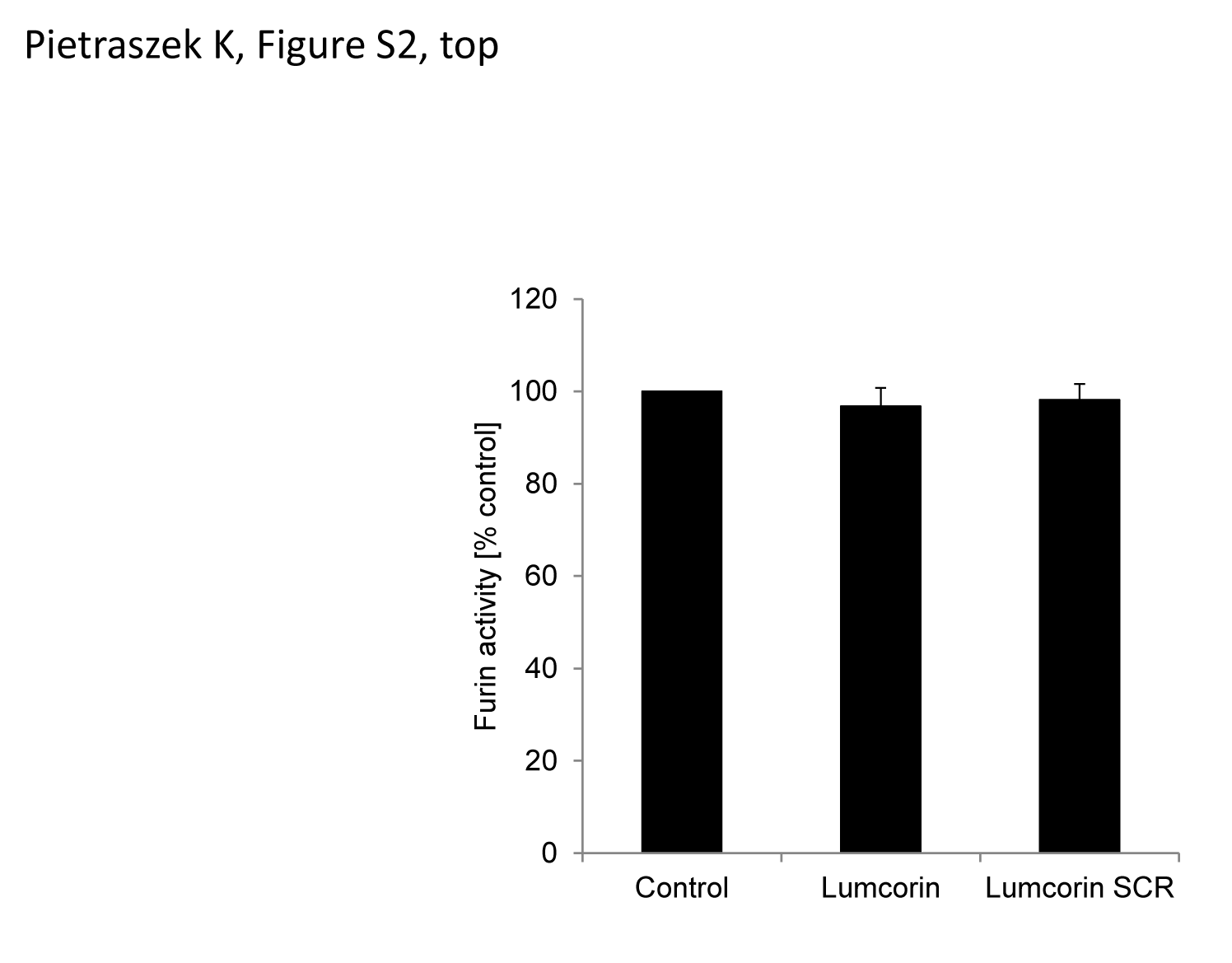

Supplement: Figure S2 — Lumcorin has no effect on furin convertase activity. Furin-like enzyme activity in B16F1 cells incubated 48h in the presence of 100µM lumcorin or its scrambled peptide, measured using fluorogenic substrate (Pyr-Arg-Thr-Lys-Arg-AMC trifluoroacetate salt) as described in Materials and Methods. (TIF) [file pone.0076232.s002.tif]

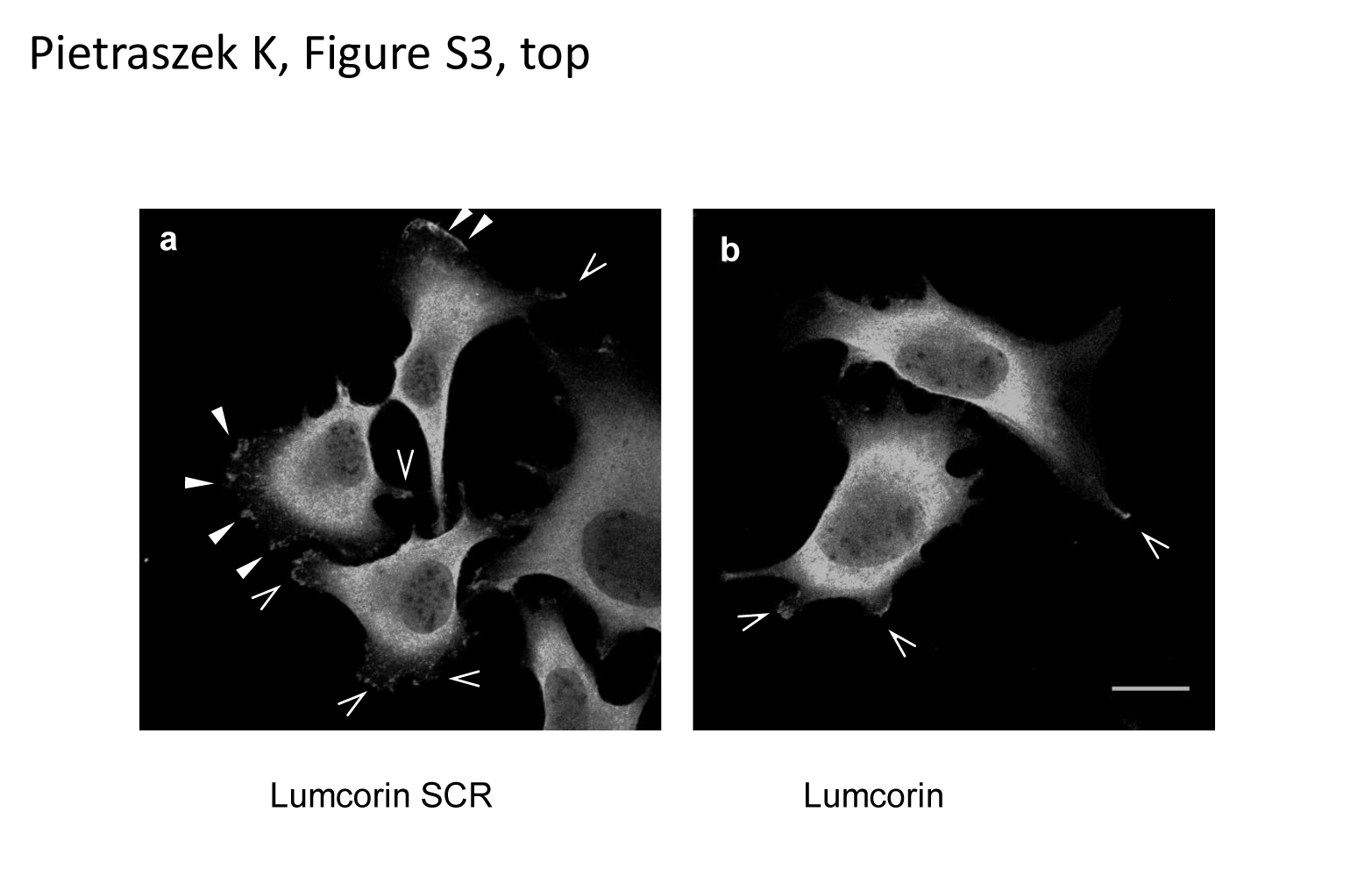

Supplement: Figure S3 — Lumcorin alters MMP-14 distribution. MMP-14 immunolocalization in B16F1 cells after 48h incubation in presence of 100µM lumcorin SCR (a), lumcorin (b). MMP-14 was visualized by confocal microscopy using an antibody directed against the hinge region of MMP-14. Arrowheads indicate the migration front and empty arrowheads focal adhesions. Scale bar: 20 µm. (TIF) [file pone.0076232.s003.tif]

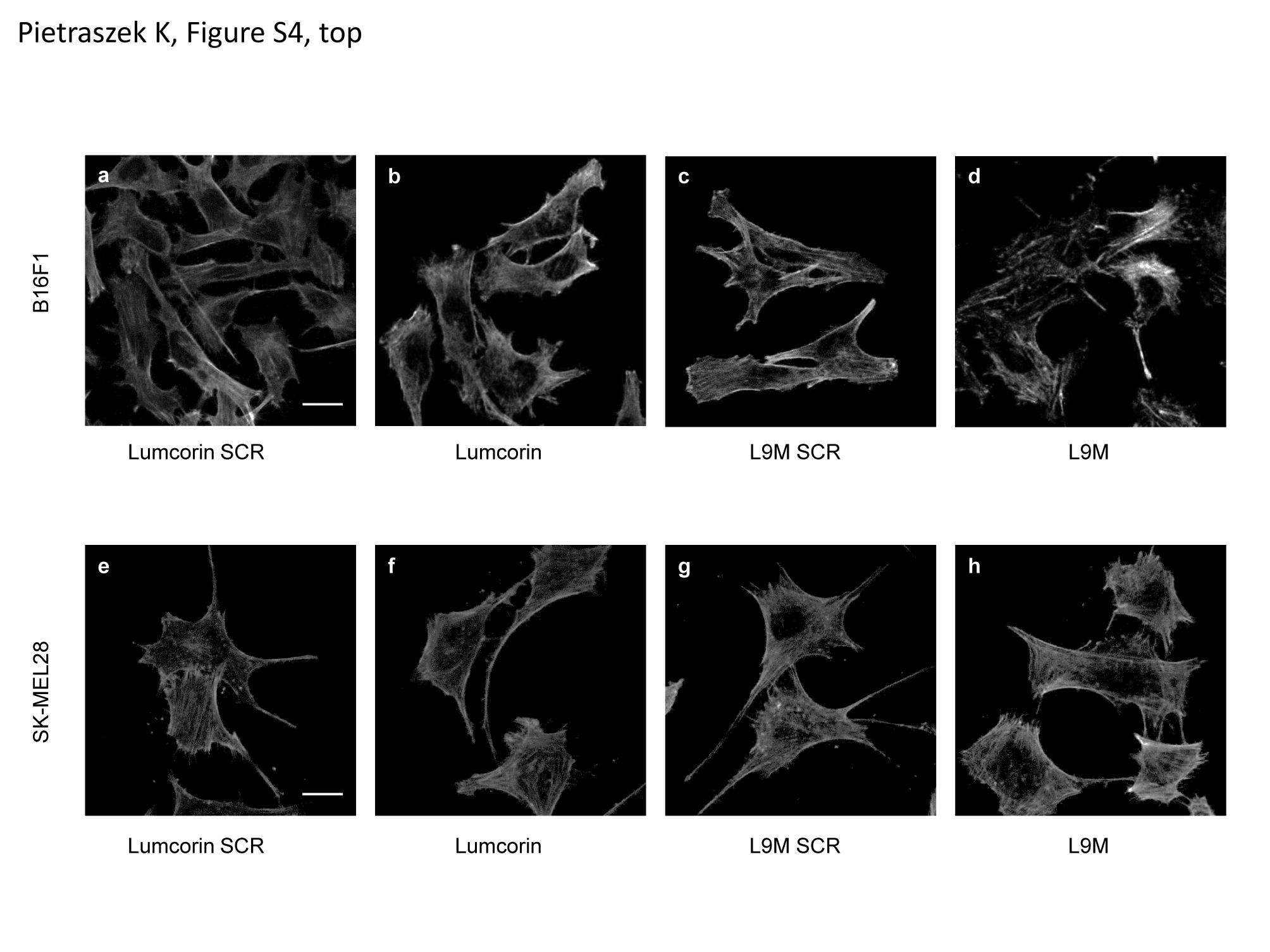

Supplement: Figure S4 — Lumcorin and L9M peptide do not induce a rearrangement of actin filament organization. Actin cytoskeleton distribution in B16F1 (a-d) and SK-MEL-28 (e-h) cells after 48h incubation in presence of 100µM lumcorin SCR (a,e), lumcorin (b,f), L9M SCR (c,g) and L9M (d,h). Filamentous actin was visualized under a fluorescence microscope after staining with Alexa Fluor ®488-conjugated phalloidin. Scale bar: 20 µm. (TIF) [file pone.0076232.s004.tif]
