# Supplementary material for: Lumican – Derived Peptides Inhibit Melanoma Cell Growth and Migration
Source: PLoS One. 2013 Oct 2;8(10):e76232. doi: 10.1371/journal.pone.0076232 (PMC3788744; doi:10.1371/journal.pone.0076232)
Supplement: File S1 — Supporting Materials and Methods. (DOC) [file pone.0076232.s005.doc]

**SUPPLEMENTAL MATERIAL**

**MATERIALS AND METHODS**

**Migration Assay**

Migration assay was performed using culture-inserts (Biovalley, Marne-la-Vallée, France). Cells were seeded on 24-well plates in culture-inserts with 3x10^4^ cells per chamber in 70 µL of complete cell culture medium. After 24h of incubation at 37°C, the culture inserts were removed, cells were rinsed with PBS and the wells were filled with 1 mL of serum-free cell culture medium supplemented with 1-100µM lumcorin or L9M and their scrambled peptides. Cell motility was followed using an inverted microscope (Axiovert 200M; Zeiss, Oberkoken, Germany) equipped with a transparent environmental chamber (Climabox; Zeiss) with 5% (v/v) CO_2_ in air at 37°C. The microscope was driven by the Metamorph® software (Roper Scientific, Evry, France). The cell position was recorded with a charge-coupled device camera (CoolsnapHQ: Roger Scientific) for 48h at 30 min intervals. Cell migration from 4 fields per insert, 3 replicate inserts for each condition, was analyzed using Image Tool software by quantification of cell-free surface and next calculated as percent of filled area.

**Quantitative Real Time PCR**

Total RNA was isolated using Tri Reagent® (Molecular Research Center, Inc. Cincinnati, Ohio, USA) according to manufacturer's protocol. The RNA quality was checked on an Agilent 2100 Bioanalyzer (Agilent Technologies, Massy, France) using the RNA 6000 Nano Assay according to the manufacturer’s instructions. Reverse transcription was performed using Maxima™ First Strand cDNA Synthesis Kit (Fermentas GmbH, Villebon sur Yvette, France) with 1 μg of total RNA. Real-time PCR experiments were done using SYBR® Green I as the intercalating agent in 96 well plates. Each 25 μL PCR mix contained cDNA template, SYBR® *Premix ExTaq*™ (TaKaRa), ROX® Reference Dye II and 0.2 μM of each gene-specific primer. PCR was run on Mx3005P thermocycler (Agilent Technologies). The following pairs of primers were used: MMP-14 forward 5’-CGGGTGAGGAATAACCAAGT-3’ and reverse 5’-CCAGAAGAGAGCAGCATCAA-3’, EEF1A1 forward 5’-CTGGAGCCAAGTGCTAATATGCC-3’ and reverse 5’-CCAGGCTTGAGAACACCAGTC-3’, GAPDH forward 5’-ACGGATTTGGTCGTATTGGG-3’ and reverse 5’-TGATTTTGGAGGGATCTCGC-3’. The specificity of PCR amplification products was assessed by dissociation melting-curve analysis. After the reaction was completed, Ct value was calculated from the amplification plots. The standard curves were generated with serially diluted solutions (1/5 – 1/3125) of cDNA from B16F1 cells. Each sample was normalized simultaneously to EEF1A1 and GAPDH housekeeping gene transcript contents. The ΔΔCt method was used for the relative quantification. PCR assays were conducted in triplicate for each sample.

**Gelatin Zymography**

Gelatin zymography was performed as previously described [1]. Cell-conditioned media were analyzed. Cells were seeded on 6-well plates in complete medium. After 24h of incubation, the culture medium was replaced by serum free medium supplemented with 100µM lumcorin or L9M and their scrambled peptides. After 48h of incubation, medium was collected and analyzed on SDS-polyacrylamide gels containing 1 mg/mL gelatin. Recombinant MMP-2 and MMP-9 (Millipore, Billerica, MA, USA) were used as markers. The gels were stained with Coomassie Brillant Blue G-250 (Sigma).

**Furin-like enzyme activity assay**

Preparation of cell lysates and furin activity assay were performed as already described [2]. B16F1 cells after 48h incubation with lumcorin or its scrambled peptide were washed twice in ice cold Dulbecco's PBS and then incubated on ice for 10 min in 5× lysis/reaction buffer (500 mM HEPES, pH 7.0, 2.5% Triton X-100, 5 mM calcium chloride, 5 mM β-mercaptoethanol). Hundred μl of 5× lysis/reaction buffer was used per 1×10^6^ cells. Furin activity was determined using 1mM furin fluorogenic substrate (Pyr-Arg-Thr-Lys-Arg-AMC trifluoroacetate salt, Bachem AG, Bubendorf, Switzerland). The activity was measured at excitation and emission wavelengths of 355 and 460 nm, respectively.

# Scanning Laser Confocal Microscopy

Immunocytochemistry was performed after 48h of incubation of B16F1 and SK-MEL-28 cells with 100µM peptides, as already described [3]. For the detection of MMP-14, a polyclonal antibody raised against the hinge region of human MMP-14 (Abcam, Cambridge, UK) was used. For the detection of actin cytoskeleton, cells were permeabilized with 0.1 % Triton X-100 and incubated 1h at room temperature with Alexa Fluor®488-conjugated phalloidin. Slides were observed under confocal laser scanning microscope (Zeiss LSM 700).

REFERENCE LIST

1. Toth M, Sohail A, Fridman R (2012) Assessment of gelatinases (MMP-2 and MMP-9) by gelatin zymography. Methods Mol Biol 878: 121-135.

2. Bourne GL, Grainger DJ (2011) Development and characterisation of an assay for furin activity. J Immunol Methods 364: 101-108.

3. Brezillon S, Radwanska A, Zeltz C, Malkowski A, Ploton D, et al. (2009) Lumican core protein inhibits melanoma cell migration via alterations of focal adhesion complexes. Cancer Lett 283: 92-100.
